# Web-Based Interventions for Weight Loss or Weight Loss Maintenance in Overweight and Obese People: A Systematic Review of Systematic Reviews

**DOI:** 10.2196/jmir.6972

**Published:** 2017-06-26

**Authors:** Angela Sorgente, Giada Pietrabissa, Gian Mauro Manzoni, Federica Re, Susan Simpson, Sara Perona, Alessandro Rossi, Roberto Cattivelli, Marco Innamorati, Jeffrey B Jackson, Gianluca Castelnuovo

**Affiliations:** ^1^ Psychology Research Laboratory, Ospedale San Giuseppe, IRCCS, Istituto Auxologico Italiano Verbania Italy; ^2^ Department of Psychology, Catholic University of the Sacred Heart Milan Italy; ^3^ eCampus University Faculty of Psychology Como Italy; ^4^ University of South Australia School of Psychology, Social Work & Social Policy Adelaide Australia; ^5^ European University of Rome Rome Italy; ^6^ Skinner Institute Rome Italy; ^7^ Virginia Tech Falls Church, VA United States

**Keywords:** Internet, review, delivery of health care, obesity, overweight, telemedicine, weight loss, body weight maintenance, treatment outcome

## Abstract

**Background:**

Weight loss is challenging and maintenance of weight loss is problematic. Web-based programs offer good potential for delivery of interventions for weight loss or weight loss maintenance. However, the precise impact of Web-based weight management programs is still unclear.

**Objective:**

The purpose of this meta-systematic review was to provide a comprehensive summary of the efficacy of Web-based interventions for weight loss and weight loss maintenance.

**Methods:**

Electronic databases were searched for systematic reviews and meta-analyses that included at least one study investigating the effect of a Web-based intervention on weight loss and/or weight loss maintenance among samples of overweight and/or obese individuals. Twenty identified reviews met the inclusion criteria. The Revised Assessment of Multiple SysTemAtic Reviews (R-AMSTAR) was used to assess methodological quality of reviews. All included reviews were of sufficient methodological quality (R-AMSTAR score ≥22). Key methodological and outcome data were extracted from each review.

**Results:**

Web-based interventions for both weight loss and weight loss maintenance were more effective than minimal or control conditions. However, when contrasted with comparable non-Web-based interventions, results were less consistent across reviews.

**Conclusions:**

Overall, the efficacy of weight loss maintenance interventions was stronger than the efficacy of weight loss interventions, but further evidence is needed to more clearly understand the efficacy of both types of Web-based interventions.

**Trial Registration:**

PROSPERO 2015: CRD42015029377; http://www.crd.york.ac.uk/PROSPERO/display_record.asp? ID=CRD42015029377 (Archived by WebCite at http://www.webcitation.org/6qkSafdCZ)

## Introduction

Obesity and overweight have reached epidemic proportions globally and pose a major risk for serious chronic diseases, including type 2 diabetes, cardiovascular disease, hypertension, sleep apnea, osteoarthritis, and certain forms of cancer [1]. Such conditions may further impact individuals’ quality of life and well-being [2]. Moreover, people suffering from weight disorders are at greater risk of social, emotional, and psychological problems such as depression, poor self-esteem, and social isolation [3]. Functional interventions aimed at reducing weight and maintaining weight loss, while working on related pathologies, are typically combined treatment options (nutritional, physical, behavioral, psychological, pharmacological, surgical) [4]. Although these usually lead to short-term weight loss, long-term maintenance of results is rarely achieved [5,6]. Consequently, alternative integrative programs aimed at supporting long-lasting weight loss are typically needed. As a result, a number of Web-based interventions for weight loss or weight loss maintenance have been recently developed, and their efficacy has been tested in a number of randomized controlled trials (RCTs). Web-based therapy could help patients overcome barriers to treatment such as long distances to clinics and long waiting times. Most Web-based interventions have zero waiting time, and all are considerably cheaper than face-to-face therapy, enabling widespread dissemination of treatment [7]. Furthermore, Web-based interventions are cost-effective and provide greater user access, flexibility, and anonymity [8]. Therefore, Web-based interventions are especially relevant for patients who might not otherwise access treatment for reasons such as fear of social stigma associated with seeking treatment.

The published systematic reviews and meta-analyses of Web-based interventions for weight loss and weight loss maintenance reveal conflicting conclusions. Thus, the purpose of this meta-review was to (1) examine the published systematic reviews that included at least one study assessing the efficacy of a Web-based intervention for weight loss and/or weight loss maintenance for samples of participants who are either overweight or obese, (2) produce a summary of the scientific evidence, (3) identify the strengths and weaknesses of Web-based interventions to help clinicians select the best treatment option for their patients, and (4) provide empirically supported suggestions for practice.

## Methods

This review was carried out according to the guidelines proposed by Smith et al [9]. The protocol for this study was registered in 2015 in the International Prospective Register of Systematic Reviews (PROSPERO).

### Inclusion and Exclusion Criteria

Given the absence of an established standard definition for systematic reviews, the following inclusion and exclusion criteria provide the parameters used for defining systematic reviews for this meta-review. Only reviews that satisfied the following criteria were included: (1) used a systematic review method (eg, critical review, literature review, meta-analysis), (2) indicated the method for identifying and evaluating studies for inclusion, (3) included at least one study assessing the efficacy of a Web-based intervention for weight loss and/or weight loss maintenance on the absolute variation and/or the change in percentage of body weight or body mass index (BMI) for a sample of overweight and/or obese people, and (4) received a methodological quality score of 22 or higher on the Revised Assessment of Multiple SysTemAtic Reviews (R-AMSTAR; see methodological quality assessment section for details). There were no restrictions for participant age, publication year, or publication language to obtain the maximum number of reviews possible. Non-English publications were translated to facilitate data extraction.

### Search Methods

As suggested by Smith et al’s guidelines [9], the following electronic databases were searched: PubMed, the Cochrane Library, PsycINFO (ProQuest platform), and the Centre for Review and Dissemination (CRD), which includes the Database of Abstracts of Reviews of Effects (DARE). Search terms were identified for each of the following relevant categories: population (obese, obesity, overweight), intervention (online, Web, computer), outcome (weight loss, weight loss maintenance), and review type (review, meta-analysis). Boolean searches were then conducted to systematically link the various combinations of category terms (and their variations through truncation) as search terms, Medical Subject Headings (MeSH) keywords and Emtree keywords to identify potential systematic reviews [10]: (“Review”[MeSH]) OR (“Meta-Analysis”[MeSH]) OR review OR meta-analysis AND (“Computers”[MeSH]) OR online OR web OR computer AND (“Weight Loss”[MeSH]) OR weight loss OR weight loss maintenance AND (“Obesity”[MeSH]) OR (“Overweight”[MeSH]) OR obese OR obesity OR overweight.

In addition, the contents of *Obesity Reviews*, *Annual Review of Public Health*, and the *Journal of Medical Internet Research* were searched using the following syntax: (review OR meta-analysis) AND (online OR web OR computer) AND (“weight loss”) AND (obes* OR overweight).

As a supplement to electronic searching, reference lists were checked to identify additional potential systematic reviews. The search was performed for records published through December 2015.

### Selection Process

Titles and abstracts of records resulting from the literature search were independently screened by authors FR and SP. When further clarification was needed, the full text was retrieved. Disagreements were resolved by a third author (AS). In accordance with one of Smith et al’s recommendations [9], the review team included at least one person with methodological expertise in conducting systematic reviews (GMM and AS) and at least two experts on the topic under review (GC, GMM, and GP).

### Data Extraction and Management

Authors SP and FR independently extracted the following data and resolved any disagreements in consultation with a third author (AS): (1) authorship and publication-related information; (2) aims of the review; (3) searched databases; (4) inclusion criteria; (5) number of included studies; (6) overall sample size and participant age, gender, race, and BMI; (7) overall length of treatment, including follow-up time points; (8) country in which the interventions were developed; and (9) outcomes of the interventions.

Reviews that included studies that did not investigate the efficacy of weight loss and/or weight loss maintenance programs among obese and overweight participants were coded for the total number of included studies and the number of included studies involving treatments for weight loss and/or weight loss maintenance in a sample of obese and/or overweight persons. Additional relevant information was obtained by retrieving original studies and contacting review authors as necessary for coding purposes.

### Methodological Quality Assessment

The R-AMSTAR [11] was used to quantitatively measure the methodological quality of included systematic reviews by assessing the presence of the following 11 domains: (1) an a priori design, (2) duplicate study selection and data extraction, (3) a comprehensive literature search, (4) the use of status of publication as an inclusion criteria, (4) a list of included/excluded studies, (5) characteristics of included studies, (6) documented assessment of the scientific quality of included studies, (7) appropriate use of the scientific quality in forming conclusions, (8) the appropriate use of methods to combine findings of studies, (8) assessment of the likelihood of publication bias, and (9) documentation of conflicts of interest. Each domain’s score ranged between 1 and 4, and the R-AMSTAR total scores had a range of 11 to 44.

A total score of 22 (ie, a mean of two criteria for each item were satisfied) was required for systematic review inclusion, thus excluding low-scoring systematic reviews [11]. The authors in charge of extracting data from the selected reviews (SP and FR) also preliminarily and independently assessed the methodological quality of the contributions. A third author (AS) resolved any discrepancies.

### Data Synthesis

First, reviews were analyzed and relevant information was extracted and recorded. Then, the results across the different reviews were aggregated through a second-order qualitative synthesis of treatment efficacy conclusions for weight loss interventions and then for weight loss maintenance interventions. Quantitative results were recorded but no second-order overall effect was calculated from the included meta-analyses including similar sets of studies because a meta-analysis of meta-analyses is possible only if the data from individual studies have not been used in more than one meta-analysis [9]. Thus, pooled effects of overlapping reviews were only compared in order to investigate the consistency of results.

Ultimately, the strengths and weaknesses of the various Web-based interventions listed across the reviews were summarized.

## Results

### Included Reviews

**Figure 1:**
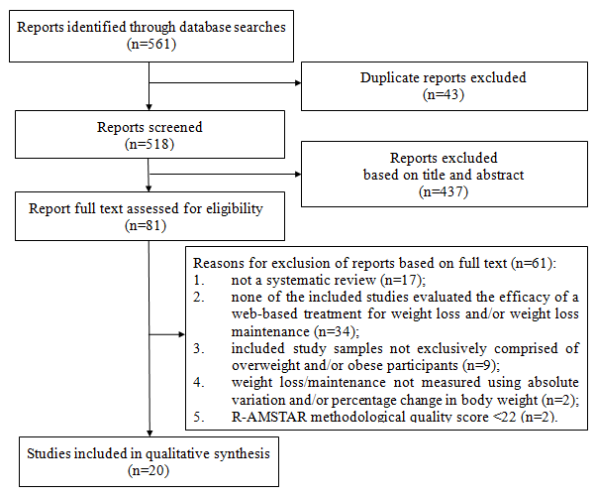
Systematic review selection flowchart.

A flowchart indicating the selection of included systematic reviews is presented in Figure 1. Searches of electronic databases identified 561 reports, of which 43 were duplicate and 437 were excluded based on information from the title and abstract. The remaining 81 reports were then evaluated for inclusion by reviewing the full text of each report, resulting in the exclusion of 61 reports for the following reasons (3 reports were omitted for more than one reason): (1) no systematic review was presented (n=17), (2) none of the included studies evaluated the efficacy of a Web-based treatment for weight loss and/or weight loss maintenance (n=34), (3) the included study samples were not exclusively comprised of overweight and/or obese participants (ie, study samples were also comprised of normal-weight participants; n=9), (4) weight change (loss or maintenance) was not measured or summarized in terms of absolute variation and/or change in percentage of body weight or BMI (n=2), and (5) the review R-AMSTAR methodological quality score was less than 22 (n=2). A total of 20 systematic reviews were finally included [12-31]. Multimedia Appendix 1 details the reasons for exclusion and whether inclusion or exclusion was based on information from the title and abstract or full text for each of the evaluated reports.

### Description of Included Systematic Reviews

Tables 1 and 2 provide a summary of the characteristics of each included systematic review. Overall, 10 [12-21] of the 20 systematic reviews examined the effects of Web-based interventions for weight loss and/or weight loss maintenance, whereas the other 10 systematic reviews [22-31] examined the effects of both Web-based and traditional interventions for weight loss and/or weight loss maintenance.

Inclusion criteria for the majority of the systematic reviews consisted of age restrictions (only adults [12-18,20,22-24,26,27,31]), research design restrictions (only RCT [12-17,19,21,24,28-30]), and outcome restrictions (differences in weight loss or weight loss maintenance as primary treatment effects [12,13,16-20,26,29,31]). In terms of other age-related inclusion criteria, five systematic reviews had no age restriction [19,21,25,29,30] and only one restricted inclusion to participants younger than 18 years [28]. In terms of BMI score inclusion criteria, with the exception of two systematic reviews that restricted study inclusion to participants with a BMI greater than 28 [26] and 30 or greater [29], the BMI cut-off was 25 or greater in the other 18 systematic reviews.

**Table 1:** Characteristics of the included systematic reviews (N=20).

Author(s), publication year	Aim of the review	Searched databases^a^	Inclusion criteria of studies	Included (relevant) studies, n (n)
Tsai et al, 2005 [22]	Describe the components, costs, and efficacy of weight loss programs	Medline	Only adults, in USA, ≥1-year follow-up assessment(s), ≥10 participants and treatment stated and program lasted ≥12 weeks	10 (1)
Weinstein, 2006 [12]	Describe the efficacy of Web-based weight loss and weight loss management programs	PubMed, CINAHL, CL, NIHCT	Only adults, in USA, RCT, ≥1 Web-based intervention, BMI≥25, published in peer-reviewed journal, primary outcome weight loss or weight loss management	8 (8)
Sharma, 2007 [23]	Review behavioral interventions for prevention and treatment of overweight and obesity	Medline	Only adults, English, published 2000-2006, educational approach	23 (2)
Neve et al, 2009 [14]	Assess the effectiveness of Web-based interventions for weight loss and weight loss management	CL, Medline, EMBASE, CINAHL, Web of Science, Scopus, PsycINFO	Only adults, RCT, ≥1 Web-based intervention, BMI≥25	18 (18)
Osei-Assibey et al, 2010 [24]	Evaluate the effectiveness of dietary and lifestyle weight loss interventions	Medline, EMBASE, CCTR, CINAHL, DARE	Only adults, RCT, ≥50% of participants were minorities, treatment lasted ≥6 months	19 (1)
Arem and Irwin, 2011 [13]	Summarize the state of the science of Internet-delivered weight loss interventions and highlight their strengths and weaknesses	PubMed	Only adults, RCT, BMI≥25, primary outcome weight loss or weight loss management, website or Web-based programming	9 (9)
Burke et al, 2011 [25]	Evaluate the effect of self-monitoring diet, physical activity level, and weight management program on weight loss in behavioral treatment studies	Medline, PsycINFO	In USA, published 1989-2009, studies on effect and use of self-monitoring	22 (3)
Manzoni et al, 2011 [16]	Evaluate the effectiveness of Web-based interventions for weight loss and weight loss management	PubMed, PsycINFO, CL, NIH	Only adults, RCT, published in peer-reviewed journal, primary outcome weight loss or weight loss management	25 (25)
Kodama et al, 2012 [15]	Review the weight loss or weight loss management effect of the Internet component in obesity treatment programs	Medline, EMBASE	Only adults, RCT, BMI≥25, website or Web-based programming	23 (23)
Reed et al, 2012 [17]	Evaluate the impact of computer-based technology on interventions for weight loss	Medline, CC, CINAHL, PsycINFO	Only adults, RCT, BMI≥25, used computer/interactive technologies, primary outcome weight loss or weight loss management, control group received non-computer-based intervention	11 (11)
Wieland et al, 2012 [18]	Assess the effect of interactive computer-based interventions for weight loss or weight loss management	CC, Medline, EMBASE, CINAHL, LILACS, PsycINFO	Only adults, BMI≥25, includes RCTs or quasi-RCTs, primary outcome weight loss or weight loss management, website or Web-based programming, lasted ≥4 weeks	18 (18)
Young et al, 2012 [26]	Investigate the effectiveness of weight loss and weight loss management interventions and identify the characteristics associated with effectiveness	CINAHL, EMBASE, Medline, PsycINFO, PubMed, Sport Discus, Scopus, Web of Science	Only adults, BMI>28, primary outcome weight loss or weight loss management, only male participants	24 (6)
Chang et al, 2013 [19]	Describe the use and impact of social media in online weight management program	PubMed, PsycINFO, EMBASE, Web of Science, Scopus	RCT, published in peer-reviewed journal, primary outcome weight loss or weight loss management, social media component	20 (20)
Grunenberg et al, 2013 [21]	Investigate the effectiveness of Web-based psychological interventions for weight loss	Medline, PsycINFO, Psyndex	RCT, BMI≥25, primary outcome weight loss or weight loss management, website or Web-based programming, control either waitlist or standard waiting treatment, psychologically based intervention for behavioral modification	5 (5)
Bennett et al, 2014 [20]	Evaluate the efficacy of eHealth weight management programs	PubMed, EMBASE, CINAHL, CL, Web of Science	Only adults, in USA, English, BMI≥25, primary outcome weight loss or weight loss management, used computer/interactive technologies	6 (6)
Hartmann-Boyce et al, 2014 [27]	Determine the clinical effectiveness of multicomponent behavioral weight management program	BIOSIS, CL, CC, CP, DR, EMBASE, HT, Medline, PsycINFO, SCI	Only adults, BMI≥25	8 (1)
Altman and Wilfley, 2015 [28]	Evaluate the evidence for overweight and obesity treatments	PubMed, PsycINFO, Google Scholar	RCT, treated children and adolescents, estimated costs for childhood obesity treatments	9 (1)
Gilmartin and Murphy, 2015 [29]	Evaluate the effectiveness of behavioral weight loss management interventions	CL, Medline, EMBASE, PsycINFO, Web of Science	RCT, primary outcome weight loss or weight loss management, BMI≥30	13 (1)
Levine et al, 2015 [30]	Examine technology-assisted weight loss interventions and highlight innovation, impact, and pragmatism	PubMed, Medline, EMBASE, CD, CC	RCT, used computer/interactive technologies, ambulatory setting	16 (8)
Raaijmakers et al, 2015 [31]	Evaluate the effectiveness of technology-based interventions on weight loss and quality of life	PubMed, PsycINFO, Web of Science, Science Direct, CINAHL, EMBASE	Only adults, BMI≥25, used computer/interactive technologies, primary outcome weight loss or weight loss management	27 (12)

^a^ BIOSIS: BIOSIS Preview; CC: Cochrane Central; CCTR: Centre for Care Technology Research; CD: Cochrane Database of Systematic Reviews; CINAHL: Cumulative Index to Nursing and Allied Health Literature; CL: Cochrane Library; CP: Cochrane Public Health Group and Evidence for Policy and Practice Information Centre; DARE: Database of Abstracts of Reviews of Effects; HT: Health Technology Assessment database; DR: Database of Abstracts of Reviews and Effects; LILACS: Latin American and Caribbean Health Sciences Literature; NIH: The National Institutes of Health; NIHCT: National Institutes of Health Clinical Trials database; RC: review of company Web sites; SCI: Science Citation Index.

**Table 2:** Characteristics of the included systematic reviews (continued) (N=20).^a^

Author, publication year	N	Age (years)	Women, %	BMI	Duration of the intervention, mean or range^b^	Country (race)^c^	Outcomes
Tsai et al, 2005 [22]	1877	—	—	—	12 wk-2 y	US	Weight
Weinstein, 2006 [12]	791	30-62	55		22 wk-12 mo	US	Weight
Sharma, 2007 [23]	—	—	—	—	3 mo-9 y	AU, BE, CN, FI, IT, JP, NL, SE, UK, US	BMI, weight, waist circumference, body fat
Neve et al, 2009 [14]	5700	≥18	77	≥25	6 wk-2 y	UK, US	Weight
Osei-Assibey et al, 2010 [24]	—	Mean=47.2	—	—	>6 mo	Western countries (people of color)	BMI
Arem and Irwin, 2011 [13]	—	34-54	50-100	Mean=29	3-18 mo	(White but 2 studies did not report race)	Weight
Burke et al, 2011 [25]	9668	—	41-100	—	—	US (>white)	
Manzoni et al, 2011 [16]	8324	≥18	76.7	—	6 wk-2 y	US and other unspecified countries	Weight
Kodama et al, 2012 [15]	8697	≥18	66.1	26.2-35.7	3-30 mo	—	Weight
Reed et al, 2012 [17]	1866	≥18	71.64	—	2-12 mo	—	Weight
Wieland et al, 2012 [18]	4140	≥18	73	>25	4 wk-30 mo	—	Weight
Young et al, 2012 [26]	1869	18-65	>0	>28	3-24 mo	AU, CN, FI, JP, NL, SE, UK, US	Weight
Chang et al, 2013 [19]	—	—	—	—	—	AU, CN, UK, US	Waist circumference, BMI, physical activity level, dietary intake
Grunenberg et al, 2013 [21]	727	≥18	57	≥25	3-12 mo	—	Weight, BMI, waist circumference
Bennett et al, 2014 [20]	4899	≥18	—	≥25	3-30 mo	US (people of color)	Weight
Hartmann-Boyce et al, 2014 [27]	>3700	40-52	—	25 (≥23 among Asians)	—	AU, CH, DE, UK, US	Weight
Altman and Wilfley, 2015 [28]	—	6-18	—	—	—	(White/people of color)	Weight
Gilmartin and Murphy, 2015 [29]	—	>18	—	≥30	>2 y	CN, FI, SE, UK, US	Weight
Levine et al, 2015 [30]	6786	Middle-aged	62	—	3-36 mo	(71% White)	Weight
Raaijmakers et al, 2015 [31]	—	—	—	>20, ≥30, ≥40	—	AU, CN, DE, JP, UK, US	Weight, quality of life, adherence

^a^ —: Information that was not reported.

^b^ Mo: month; wk: week; y: year.

^c^ AU: Australia; BE: Belgium; CH: Switzerland; CN: China; DE: Germany; FI: Finland; IT: Italy; JP: Japan; NL: Netherlands; SE, Sweden; UK: United Kingdom; US: United States.

The total number of participants in each systematic review ranged from 727 [21] to 9668 [25]. In terms of gender, nine systematic reviews reported no gender-specific data [32-36]; the majority of reviews that included information on participant gender had samples with more female participants than male participants.

The number of databases that were searched for each systematic review ranged from 1 [13,23] to 10 [27]. A total of 351 studies were evaluated across the 20 systematic reviews, of which only 83 evaluated the effects of Web-based interventions for weight loss and/or weight loss maintenance. Of the 83 studies, 73 evaluated Web-based interventions for weight loss and 10 evaluated Web-based interventions for weight loss maintenance (see Multimedia Appendix 2 for details). The 83 studies were mostly conducted in the United States, Europe, or Australia; 51 of 83 studies were only included in one systematic review and the remaining 32 studies were included in more than one systematic review. The study that was included in the most systematic reviews [37] was included in a total of eight reviews.

### Methodological Quality of Included Reviews

The R-AMSTAR scores of the 20 included reviews (Table 3) ranged from 23 to 43 points, with a mean of 30.5 (SD 5.5), a median of 30.5 (IQR 9.25). The highest mean score across the 20 systematic reviews (mean 4, SD 0) was for providing the characteristics of the included studies (item 6), whereas the lowest mean score was for the inclusion of conflicts of interest (item 11; only Hartmann-Boyce et al [27] fully satisfied this criterion).

**Table 3:** Systematic review quality (N=20).

Systematic review	R-AMSTAR Item^a^											Score
	1	2	3	4	5	6	7	8	9	10	11	
Tsai et al, 2005 [22]	3	4	3	1	3	4	4	4	1	1	1	29
Weinstein, 2006 [12]	3	1	4	2	4	4	1	2	1	1	1	24
Sharma, 2007 [23]	3	4	1	3	3	4	1	1	1	1	1	23
Neve et al, 2009 [14]	4	4	4	1	3	4	4	4	4	4	1	37
Osei-Assibey et al, 2010 [24]	3	4	4	3	2	4	4	4	2	1	1	32
Arem and Irwin, 2011 [13]	3	4	4	2	3	4	1	1	1	1	1	25
Burke et al, 2011 [25]	3	1	4	4	3	4	1	1	1	1	1	24
Manzoni et al, 2011 [16]	3	4	4	1	3	4	1	1	1	1	1	24
Young et al, 2011 [26]	3	4	4	3	4	4	4	4	2	1	1	34
Kodama et al, 2012 [15]	3	4	3	1	1	4	1	1	4	4	1	27
Reed et al, 2012 [17]	4	4	4	1	3	4	4	1	4	4	1	34
Wieland et al, 2012 [18]	4	4	4	4	4	4	4	1	4	4	1	38
Chang et al, 2013 [19]	4	4	4	3	3	4	4	4	1	1	1	33
Grunenberg et al, 2013 [21]	3	1	3	2	1	4	4	4	4	2	3	31
Bennett et al, 2014 [20]	3	4	4	4	4	4	4	4	1	1	1	34
Hartmann-Boyce et al, 2014 [27]	4	4	4	3	4	4	4	4	4	4	4	43
Altman and Wilfley, 2015 [28]	3	4	3	1	3	4	1	1	2	1	1	24
Gilmartin and Murphy, 2015 [29]	3	4	3	2	3	4	4	4	1	1	1	30
Levine et al, 2015 [30]	4	4	4	4	3	4	1	1	4	4	1	34
Raaijmakers et al, 2015 [31]	3	4	4	1	3	4	4	4	1	1	1	30
Mean	3.32	3.55	3.60	2.30	3.00	4.00	2.80	2.55	2.20	1.95	1.25	30.50
Median	3.00	4.00	4.00	2.00	3.00	4.00	4.00	3.00	1.50	1.00	1.00	30.50
SD	0.48	1.10	0.75	1.17	0.86	0.00	1.51	1.50	1.40	1.39	0.79	5.54
IQR	1	0	0.5	2	0.25	0	3	3	3	3	0	9.25

^a^ Item 1: a priori design; item 2: duplicate study selection and data extraction; item 3: comprehensive literature search; item 4: publication status as an inclusion criteria; item 5: list of included and excluded studies; item 6: characteristics of included studies; item 7: documented assessment of the scientific quality of included studies; item 8: appropriate use of the scientific quality in forming conclusions; item 9: appropriate use of methods to combine study findings; item 10: assessment of publication bias likelihood; item 11: conflict of interest documentation.

### Efficacy of Web-Based Interventions for Weight Loss and/or Weight Loss Maintenance

Effect sizes of Web-based interventions for weight loss and weight loss maintenance, together with the specific comparison interventions, are reported in Table 4. The intervention purpose (ie, weight loss or weight loss maintenance) is also specified in Table 4. In addition, details for each meta-analysis, such as the number of studies used to calculate effect sizes, the heterogeneity among included studies, and the combined sample size are also reported in Table 4. Except for Kodama et al [15], all meta-analyses performed quantitative data synthesis separately for both the type of condition compared to the Web-based intervention and whether the purpose of the intervention was weight loss or weight loss maintenance. Given that several primary studies were included in more than one meta-analysis, issues related to statistical independence prevented meta-meta-analysis of the effect sizes across the meta-analyses. Overall, the meta-analysis effect sizes were relatively small in magnitude, suggesting that although Web-based interventions were significantly more or less effective than the comparison conditions, this difference may have little clinical relevance.

### Web-Based Interventions for Weight Loss

#### Web-Based Interventions Versus Control Conditions (Minimal Interventions)

Across reviews, Web-based interventions were found to be significantly more effective than minimal treatments in reducing weight. Specifically, Wieland et al [18] found that Web-based interventions were significantly more effective than minimal treatments in reducing weight and BMI at 3- and 6-month follow-ups. Young et al [26] and Weinstein [12] also obtained a signiﬁcant difference in weight change favoring Web-based interventions over controls. Additionally, in Raaijmakers et al’s review [31], six technological-based interventions generated a significantly greater effect in terms of weight loss than no treatment conditions. In Bennett et al’s review [20], more than half of the identiﬁed trials reported signiﬁcantly greater weight loss outcomes for eHealth interventions compared to control conditions. Similar results were found in Levine et al’s review [30], in which 12 Web-based interventions (75%) resulted in greater weight loss compared to control conditions. Finally, Grunenberg et al [21] found a Web-based intervention to be more effective than control groups (waitlist and standard waiting treatment) at reducing both BMI and weight. Neve et al [14] was the only review to report no signiﬁcant difference in weight loss between Web-based interventions and control groups at treatment termination. This contrasting finding may be attributable to Neve et al [14] including fewer studies that tested this comparison (n=3) than the other reviews that found Web-based intervention to be more effective at promoting weight loss compared to control conditions. Also, the meaning of the term “control condition” varied across reviews from no intervention [31] to providing participants with a weight loss manual [14]. Additionally, Neve et al [14] combined treatment effects irrespective of time points (from 16 weeks to 12 months), whereas other reviews pooled the studies’ effects separately for each follow-up point.

#### Web-Based Interventions Versus Non-Web-Based Comparable Interventions

Included systematic reviews that included studies comparing Web-based treatments with non-Web-based comparable interventions presented inconsistent results. For example, Raaijmakers et al [31] found Web-based interventions to be more effective than usual care, and Tsai et al [22] found greater weight reduction among the participants assigned to a Web-based condition (ie, Weight Watchers) than those receiving self-help interventions. Levine et al [30] also concluded that technology-based interventions can successfully supplement primary care interventions for weight loss outcomes. Finally, Weinstein [12] found that Web-based interventions are significantly more effective than their non-Web-based counterparts both when the latter consists of usual care or when participants receive information from a manual.

On the other hand, other reviews found Web-based interventions to be as effective as non-Web-based comparable interventions. Specifically, Burke et al [25] examined three studies on online dietary self-monitoring and found that online treatments resulted in significant within-group weight loss; however, when compared with a paper diary self-monitoring condition, the pooled effect size was no longer statistically significant. In addition, Bennett et al [20] found that eHealth approaches led to relatively modest weight loss outcomes with undetermined clinical signiﬁcance when compared with traditional individual and group-based interventions. Finally, Reed et al [17] found that computer-based technology led to significantly less weight loss than comparable interventions. Therefore, the research on the efficacy of Web-based interventions compared to similar non-Web-based interventions is inconclusive. This lack of consistency may be due to the large heterogeneity of non-Web-based comparison interventions in the primary studies. For example, the non-Web-based comparison interventions ranged from manualized interventions to a counseling program in the studies included by Raaijmakers et al [31].

**Table 4:** A summary of meta-analyses.

Review and comparison^a^		Number of included articles	Outcome (units) and follow-up	N	Heterogeneity^b^				Effect size^c^ (95% CI)	*P*
					χ^2^ (df)	*P*	T^2^	I^2^		
**Neve et al, 2009 [14]**										
	Web vs control	3	Weight loss (kg)	151	12.8 (2)	.002	—	84.4%	0.73 (–0.6, 1.51) WMD	.07
	Enhanced Web vs basic Web	3	Weight loss (kg)	217	3.8 (3)	.28	—	21%	2.24 (1.27, 3.21)^d^ SMD	<.001
	Web vs control	2	Weight loss maintenance (kg)	409	0.02 (1)	.90	—		–0.30 (–0.34,–0.26)^d^ WMD	<.001
	Web vs face-to-face	2	Weight loss maintenance (kg)	182	12.2 (3)	.007	—	76%	1.80 (–1.18, 4.79) WMD	.24
Kodama et al, 2012 [15]		23	Weight loss, weight loss maintenance (kg)	8697	—	<.001	—	84%	–0.68 (–1.29,–0.08) WMD	.03
**Reed et al, 2012 [17]**										
	Intervention with Web vs without Web	5	Weight loss (kg)	336	0.7 (5)	.98	0.00	0%	–1.48 (–2.52,–0.43) WMD	.006
	Web vs non-Web	5	Weight loss (kg)	544	14.2 (5)	.01	3.61	65%	0.36 (–1.80, 2.53) WMD	.74
	Web vs non-Web	4 (articles published in 1995 or later)	Weight loss (kg)	538	1.7 (4)	.78	0.00	0%	1.47 (0.13, 2.81) WMD	.03
	Intervention with Web vs without Web	4	Weight loss (kg); short-term follow-up	100	0.2 (4)	>.99	0.00	0%	–1.89 (–3.41,–0.38) WMD	.01
	Intervention with Web vs without Web	1	Weight loss (kg); long-term follow-up	236	NA	NA	NA	NA	–1.10 (–2.55, 0.35) WMD	.14
	Intervention with Web vs without Web	2	Weight loss (kg); short-term follow-up	53	0.04 (1)	.85	0.00	0%	–1.95 (–3.50,–0.40) WMD	.01
	Intervention with Web vs without Web	3	Weight loss (kg) long-term follow-up	283	0.03 (3)	>.99	0.00	0%	–1.08 (–2.50,–0.34) WMD	.14
	Intervention with Web vs without Web	2 (articles published prior to 1995)	Weight loss (kg)	47	0.02 (2)	.99	0.00	0%	–0.63 (–7.91, 6.66) WMD	.87
	Intervention with Web vs without Web	3 (articles published in 1995 or later)	Weight loss (kg)	289	0.6 (2)	.72	0.00	0%	–1.50 (–2.55, 0.44) WMD	.006
	Intervention with Web vs without Web	3	Weight loss (BMI)	380	0.8 (2)	.67	0.00	0%	–0.43 (–0.83,–0.03) WMD	.04
	Web vs non-Web	2	Weight loss (BMI)	51	0.3 (2)	.88	0.00	0%	0.44 (–1.15, 2.03)^c^ WMD	.59
	Web vs control	2	Weight loss (kg)	511	0.04 (1)	.84	0.00	0%	−1.5 (−2.1, −0.9) MD	<.001
**Wieland et al, 2012 [18]**										
	Web vs control	2	Weight loss maintenance (kg)	897	0.7 (1)	.41	0.00	0%	−0.7 (−1.2, −0.2) MD	.004
	Web vs face-to-face	2	Weight loss maintenance (kg)	897	2.9 (1)	.09	0.41	66%	0.5 (−0.5, 1.6) MD	.32
**Grunenberg et al, 2013 [21]**										
	Web vs control	5	Weight loss (BMI)	727	10.5 (4)	.03	0.15	62%	–0.49 (–0.95,–0.03) MD	.04
	Web vs control	5	Weight loss (kg)	727	16.7 (4)	.002	1.46	76%	–1.32 (–2.59,–0.06) MD	.04

^a^ Web vs control: Web-based intervention vs control condition (minimal intervention); enhanced Web vs basic Web: enhanced Web-based interventions vs basic Web-based interventions; Web vs face-to-face: Web-based intervention vs face-to-face intervention; intervention with Web vs intervention without Web: adding a Web-based component to an intervention vs the same intervention without the Web-based component; Web vs non-Web: Web-based interventions vs non-Web-based comparable interventions.

^b^ Ι^2^: Percentage of the variation across studies attributable to study heterogeneity rather than chance, indicating the level of inconsistency across study results; Τ^2^: between-study variance.

^c^ Effect sizes were retrieved from original articles reporting a statistically significant pooled effect estimated from at least two trials. All studies except for those indicated used a random effects model to calculate the aggregated effect size. MD: mean difference; SMD: standardized mean difference (Cohen *d*; standardized weighted aggregated average difference score between conditions across primary studies that use different outcome measures/metrics; to facilitate aggregation across measures/metrics, the between-condition difference for each primary study is converted to standard deviation units that are then weighted with primary studies with more precise estimates carrying more weight in aggregation); WMD: weighted mean difference (unstandardized weighted aggregated average difference score between conditions across primary studies that use the same outcome measure/metric; the between-condition difference for each primary study is weighted with primary studies with more precise estimates carrying more weight in aggregation).

^d^ A fixed effect model was used to calculate the aggregated effect size.

#### Web-Based Interventions Versus Face-to-Face Interventions

This section summarizes results from systematic reviews in which Web-based interventions were compared with non-Web-based counterparts involving face-to-face interventions. In Wieland et al [18], face-to-face interventions were more effective at promoting weight loss than Web-based interventions. Also, Raaijmakers et al [31] reviewed a primary study in which face-to-face treatment led to a significantly greater reduction in weight than Web-based intervention. Similarly, Kodama et al [15] concluded that using a Web-based intervention as a substitute for a face-to-face intervention produced unfavorable results.

#### Web-Based Interventions Versus Hybrid Interventions Versus Face-to-Face Interventions

Web-based interventions were further compared with hybrid interventions (ie, including both Web-based and non-Web-based components) in several systematic reviews. For example, Kodama et al [15] came to the conclusion that adding face-to-face interventions to Web-based interventions increases the impact of the Web-based interventions on weight loss. In contrast, Wieland et al [18] reported that Web-based interventions and hybrid conditions (ie, Web-based intervention face-to-face treatment) did not differ significantly in their effects. In the study reported by Wieland et al [18], the hybrid condition was also compared with the face-to-face intervention without Web-based components. This pairwise comparison indicated that mean weight loss achieved by face-to-face treatments was significantly greater than mean weight loss achieved by hybrid conditions. In comparison, Reed et al [17] determined that computer-based treatments combined with standard interventions (ie, behavioral programs, face-to-face treatments) resulted in significantly more weight loss than standard interventions only, at least when short-term effects were considered. Similarly, Tsai et al [22] found significantly greater weight loss in participants receiving a Web-based treatment (ie, Weight Watchers program) combined with individualized contacts than in participants receiving a face-to-face intervention. Due to these contrasting results, it is not clear if hybrid interventions are more effective in increasing weight loss than single component interventions (ie, either only Web-based or only non-Web-based).

#### Enhanced Web-Based Interventions Versus Basic Web-Based Interventions

Several systematic reviews also compared the effects of Web-based interventions that differed on both the interaction level and the extent to which they were tailored to users’ needs. Osei-Assibey et al [24] and Hartmann-Boyce et al [27] reported that Web-based tailored programs were more effective in weight loss than information-only websites, despite the disappearance of this difference by 18 months after treatment [27]. Levine et al [30] concluded that interventions including clinician-guided software or feedback from personnel promoted greater weight loss than fully automated interventions, thus underlining the importance of the interactive component. Both Neve et al [14] and Wieland et al [18] were also in agreement about Web-based interventions with interactive components being effective in reducing weight. Specifically, the enhanced Web-based interventions considered in both reviews included additional programs, such as email-based behavioral therapy delivered by a doctoral-level therapist (including feedback and behavioral lessons), behavioral e-counseling provided by a counselor (weekly email behavioral counseling and feedback), and automated e-counseling (weekly automated and tailored messages). Sharma [23] reported a greater weight reduction in behavioral e-counseling conditions compared to basic Web-based programs, and Manzoni et al [16] concluded that Web-based behavioral programs enhanced by tailored feedback or self-monitoring resulted in more effective weight reduction than education-only Web-based interventions. Similarly, Osei-Assibey et al [24] found that weight change was greater for Web-based programs supporting collaborative interactions than for Web-based educational interventions. Furthermore, Weinstein [12] concluded that online counseling may be a valid alternative to time-consuming clinical programs and health care costs. Still, in Altman and Wilfley [28], an included study revealed a Web-based lifestyle behavior modification program to be more effective that a Web-based health education program at treatment termination, but not at 2-year follow-up (probably because program usage decreased over time). There was some evidence that website usage was associated with enhanced outcomes. For example, one study included in Chang et al [19] reported a Web-mediated walking program that was administered both alone and in conjunction with online community components. No differences were found in physical activity outcomes between participants who had access to social media versus those who did not; however, among participants using online communities, higher use of social media was associated with greater weight loss. Overall, these findings suggest that tailored and interactive Web-based interventions promote greater weight loss than basic Web-based interventions (ie, delivering information via the Internet). However, results also indicate that utilization of Web-based resources has potential to boost treatment effectiveness.

### Within-Subject Comparisons

Arem and Irwin’s review [13] summarized the results of studies measuring within-group effects of Web-based interventions by comparing weight outcomes before and after treatment. Findings indicate Web-based interventions caused a decrease in weight ranging from 0.8 kg (considered to be natural noise) to 4.9 kg. The authors concluded that the large degree of treatment heterogeneity across studies reduced their ability to make reliable conclusions.

### Web-Based Interventions for Weight Loss Maintenance

#### Web-Based Interventions Versus Control Conditions (Minimal Interventions)

Six reviews compared Web-based weight loss maintenance interventions with control conditions with consistent results. Specifically, Neve et al [14], Manzoni et al [16], Gilmartin and Murphy [29], Young et al [26], Bennett et al [20], and Wieland et al [18] found that Web-based interventions were, on average, significantly more effective than minimal interventions in promoting weight loss maintenance.

#### Web-Based Interventions Versus Non-Web-Based Comparable Interventions

Two systematic reviews reported results of studies comparing Web-based interventions for weight loss maintenance with non-Web-based comparable interventions. Kodama et al [15] concluded that, in comparison with non-Web-based conditions, Web-based programs were ineffective. In contrast, Bennett et al [20] reported on a study in which an interactive Web-based intervention was compared to a monthly face-to-face or telephone-based intervention. In this case, the amount of weight regained did not differ significantly between the two interventions. Overall, the inconsistent results for this particular comparison of treatments may be due to the diverse characteristics of the non-Web-based interventions that were provided.

#### Web-Based Interventions Versus Face-to-Face Interventions

Change et al [19], Weinstein [12], Neve et al [14], and Manzoni et al [16] reported that maintenance of weight loss was similar between Web-based and non-Web-based face-to-face interventions. In comparison, Gilmartin and Murphy [29] and Wieland et al [18] concluded that Web-based treatments were less effective than face-to-face interventions, especially if the latter were intensive and not minimal [12]. Specifically, Gilmartin and Murphy [29] stated that face-to-face interventions and facilitator-led interventions were more eﬀective than remotely delivered methods such as Web-based interventions. Finally, Wieland et al [18] referred to three studies comparing face-to-face interventions with Web-based interventions for weight loss maintenance. Both minimal (once monthly or less) and intensive (more than once per month) face-to-face interventions were found to be more effective than computer-based interventions. However, the amount of weight lost by persons assigned to the control (minimal) conditions was relatively small and was not maintained in the long term, making the clinical significance of these differences unclear.

### Strengths and Weaknesses of Web-Based Interventions for Weight Loss and Weight Loss Maintenance

Eleven systematic reviews provided information about the strengths and weaknesses of the evaluated Web-based interventions, with seven of them specifically identifying three advantages of Web-based interventions:

They may enhance perceived self-control within treatment. Specifically, Levine et al [30], Manzoni et al [16], Kodama et al [15], and Raaijmakers et al [31] pointed out that Web-based interventions allow people to self-monitor their weight and behaviors, thereby increasing their perceived sense of control and ultimately reducing the number of dropouts [15].

They may facilitate patient-patient and patient-expert interactions, thus allowing people to receive regular consistent feedback on their behaviors and answers to questions [16,30,31].

Web-based interventions for weight loss are more cost-effective than standard treatments [31].

Only three systematic reviews reported weaknesses associated with Web-based interventions for weight loss and weight loss maintenance. Arem and Irwin [13] reported that the limited effectiveness of Web-based interventions may be due to the restricted range of programs and updates that are available, which may not always be suitable to meet users’ needs. Bennett et al [20] and Chang et al [19] indicated that Web-based treatments may be affected by low levels of familiarity and self-efficacy associated with managing Web technologies, as well as by limitations associated with access to the Internet.

## Discussion

### Principal Results

To our knowledge, this systematic review of systematic reviews represents the first state-of-the-art analysis of Web-based intervention efficacy for weight loss and weight loss maintenance. According to the selection criteria, 20 systematic reviews were deemed eligible for inclusion. All 20 systematic reviews were published in 2005 or later. They mainly investigated Web-based interventions for weight loss, with only a few investigating Web-based interventions for weight loss maintenance. Findings from the meta-systematic review regarding Web-based interventions for weight loss and weight loss management were mixed; in fact, the findings within the included systematic reviews are often conflicting, particularly in relation to the efficacy of Web-based weight loss interventions. The conflicting results are likely due to the notable heterogeneity of inclusion criteria across the systematic reviews for selecting primary studies. Nevertheless, all the included systematic reviews demonstrated methodological rigor (R-AMSTAR score ≥22), although none received the highest possible score for methodological quality. Specifically, Hartmann-Boyce et al [27] was the only systematic review that fully met the 11th R-AMSTAR criterion of disclosing conflicts of interest, ensuring the validity of the systematic review results. Indeed, by not declaring conflicts of interest, it is impossible to rule out the existence of publication bias. The synthesis of the included systematic reviews identified both strengths and weaknesses of the Web-based interventions for both weight loss and weight loss maintenance. Web-based interventions may facilitate continuous and automated tracking of health-related behaviors by supporting self-regulatory techniques, patient involvement, and patient commitment to treatment. Moreover, Web-based connectivity permits the sharing of information among health professionals and peers. However, the efficacy and dissemination of Web-based interventions may be affected by the gap in access to computers and the Internet, as well as the lack of technological literacy among potential users.

### Limitations

In conducting this systematic review of systematic reviews, it was sometimes difficult to make a clear distinction between Web-based interventions (delivered over the Internet) and computer-based interventions (delivered over the Internet or by installing computer software) because these terms are often used interchangeably or defined differently [18]. It was also difficult to compare the overall effects across systematic reviews since they were calculated differently (ie, weighted mean difference vs standardized mean difference). Furthermore, conclusions of a second-order review are not drawn from results of primary studies, but from reviews that have synthesized the results of primary studies. Because the same primary studies were often included in more than one systematic review, not only did this overrepresentation prevent meta-meta-analysis of the efficacy of Web-based interventions for weight loss and weight loss maintenance, it also compromised the accuracy of the meta-systematic review findings, thus affecting the actual reliability of findings based on second-order data synthesis. In addition, because the number of primary studies on which each systematic review was based varied substantially (from 1 to 25), the findings from some systematic reviews were based on more evidence than the findings of other systematic reviews. Given the limitations associated with this meta-systematic review, the conclusions should be interpreted with some caution.

### Conclusion

#### Are Web-Based Interventions for Weight Loss and Weight Loss Maintenance Effective?

This systematic review of systematic reviews concludes that Web-based interventions for weight loss are often more effective than minimal treatments (only Neve et al [14] reached different conclusions); however, when compared with non-Web-based or hybrid interventions, results appear inconsistent across reviews. More encouraging results in terms of weight loss were obtained when Web-based interventions were enhanced (ie, more interactive and tailored) than when they were basic (ie, information website). Nevertheless, Web-based interventions for weight loss were less effective than face-to-face interventions across the selected reviews. Results were more encouraging in relation to Web-based weight loss maintenance interventions, which were found to be more effective than minimal interventions across all the reviews and, in some reviews, as effective as the non-Web-based counterpart. The decision of whether or not to substitute an in-person intervention for weight loss maintenance with a comparable Web-based treatment mainly depends on patient costs, needs, and preferences.

These conclusions should be considered cautiously. Reported effect sizes were small; for example, weight loss of 1 to 2 kg may not by clinically significant, irrespective of significance level. Also, conclusions might be affected by heterogeneity across primary studies. In fact, research designs differed in terms of type of intervention, sample size, duration, control condition, etc. Although the conclusions from this meta-systematic review are of significant interest, the real impact of Web-based interventions for weight loss remains unclear, suggesting the need for greater clarity in both the definition and specificity of the different types of Web-based treatments available, as well as how each intervention can be best matched to users’ needs. Further evidence is therefore necessary.

#### Suggestions and Implications for Future Research

Authors interested in providing a new summary review of the literature on the efficacy of Web-based interventions for weight loss and weight loss maintenance for obese and overweight patients can refer to the list of included records reported in Multimedia Appendix 2. A total of 83 primary studies investigating the effectiveness of online interventions for weight loss and weight loss maintenance were identified, analyzed, and compared across systematic reviews. A single study-level review of these primary studies that pinpoints differences and inconsistences across the primary studies would be beneficial. In addition, meta-analysis of these primary studies would provide a quantitative summary of the efficacy of Web-based treatments for weight loss and weight loss maintenance.

Future systematic reviews should provide a high level of detail when reporting primary study effect sizes. Specifically, detailed information about the nature of the comparison conditions (especially for instances in which there are multiple comparison conditions) and the various types of efficacy outcomes is necessary to allow other researchers and practitioners to more clearly interpret the results and to facilitate replication of these studies. Also, the effects of Web-based interventions for weight loss and Web-based interventions for weight loss maintenance should not be compared with each other [15] because they differ in both aims and outcomes. In addition, researchers and practitioners should carefully consider the cost of Web-based intervention; although technology-based treatments are fundamental in reducing health care costs, cost-effectiveness is often not adequately evaluated (if at all) in comparing Web-based and face-to-face interventions. Therefore, interventions should be compared in terms of both efficacy and cost-effectiveness (see Raaijmakers et al [31] and Wieland et al [18] for examples of reviews that evaluated treatment efficacy and cost-effectiveness).

## Appendix

Multimedia Appendix 1 Reasons for exclusion for each record.

Multimedia Appendix 2 Target studies included in the reviews.

## Abbreviations

BMI: body mass index
CRD: Centre for Review and Dissemination
DARE: Database of Abstracts of Reviews of Effects
MeSH: Medical Subject Headings
PROSPERO: Prospective Register of Systematic Reviews
R-AMSTAR: Revised Assessment of Multiple SysTemAtic Reviews
RCT: randomized controlled trial
